# The outcomes of corneal sight rehabilitating surgery in Stevens-Johnson syndrome: case series

**DOI:** 10.1186/s12886-024-03461-2

**Published:** 2024-05-06

**Authors:** Rongmei Peng, Miaomiao Chi, Gege Xiao, Hongqiang Qu, Zhan Shen, Yinghan Zhao, Jing Hong

**Affiliations:** 1 Department of Ophthalmology, Peking University Third Hospital, Beijing, China; 2 Key Laboratory of Vision Loss and Restoration, Ministry of Education, Beijing, China

**Keywords:** Stevens-Johnson syndrome, Keratoplasty, Keratolimbal allograft, Toxic epidermal necrolysis, Ocular SJS

## Abstract

**Purpose:**

To summarize the outcomes of corneal sight rehabilitating surgery in Stevens-Johnson syndrome (SJS).

**Methods:**

This is a retrospective analysis of a consecutive case series. Twenty-four eyes of 18 SJS patients were included in this study. The ocular parameters, surgical procedures, postoperative complications, and additional treatments of the cases were reviewed.

**Results:**

A total of 29 corneal sight rehabilitating surgeries, which consists of 9 keratoplasties, 8 Keratolimbal allograft (KLAL) and 12 combined surgeries (keratoplasty and KLAL simultaneously) were performed on the 24 eyes. All patients were treated with glucocorticoid eyedrops and tacrolimus eyedrops for anti-rejection treatment without combining systemic immunosuppression, except two patients who were prescribed prednisone tablets for the management of systemic conditions. The mean follow-up period was 50.6 ± 28.1 months. The optimal visual acuity (VA) (0.74 ± 0.60 logarithm of the minimum angle of resolution [logMAR]) and endpoint VA (1.06 ± 0.82 logMAR) were both significantly better than the preoperative VA (1.96 ± 0.43 logMAR) (95% CI, *p* = 0.000). 57.1% patients (8/14) were no longer in the low vision spectrum, and 88.9% patients (8/9) were no longer blind. The mean epithelialization time was 7.1 ± 7.6 weeks. The success rate was 86.7%. Additional treatments for improving epithelialization included administration of serum eyedrops (*n* = 10), contact lens (*n* = 15), amniotic membrane transplantation (*n* = 6), and tarsorrhaphy (*n* = 8). Complications included delayed epithelialization (*n* = 4, over 12 weeks), glaucoma (*n* = 11), and severe allograft opacity (*n* = 4). Only one graft rejection was observed.

**Conclusions:**

Keratoplasty and KLAL can remarkably enhance VA and improve low vision or even eliminate blindness for ocular complications of SJS. The outcome of the surgeries was correlated with the preoperative ocular situation and choice of operative methods.

**Supplementary Information:**

The online version contains supplementary material available at 10.1186/s12886-024-03461-2.

## Introduction

Stevens-Johnson syndrome (SJS) and toxic epidermal necrolysis (TEN) are acute blistering disorders of the skin and mucous membrane [1]. These disorders commonly affect the ocular surface as well [2]. In the acute stage, ocular tissues undergo necrosis and apoptosis, which leads to persistent conjunctivitis, epithelial defect, corneal ulceration, and even perforation [3]. In the chronic stage, scarring and inflammation can cause cicatricial sequelae including limbal stem cell deficiency (LSCD), corneal haze, corneal vascularization, corneal conjunctivalization, and even perforation. [4–7]. Besides, the severe dry eye makes the management of such cases difficult [8]. Therefore, SJS and TEN often lead to moderate to severe visual impairment or blindness.

When corneal blindness or corneal perforation occurs, keratoplasty is routinely performed for corneal visual rehabilitation [9, 10]. However, the treatment of the ocular surface disorders caused by SJS is still a challenge for ophthalmologists. Keratoplasty, which was used to treat these disorders, is currently considered best avoided in such cases [11]. The number of available reports on keratoplasty for the treatment of ocular complications of SJS/TEN is limited; most of them are reports of limbal stem cell transplantations [12, 13]. Most of the procedures in these reports had a low success rate, and the results were reported as part of larger ocular surface diseases series [14, 15]. Some of the procedures in these cases were combined with penetrating keratoplasty (PKP) or anterior lamellar keratoplasty (ALKP), but almost all the grafts failed because of rejection, infection, or inflammation [16–18]. Regarding corneal perforation, Wang et al. used a modified small tectonic keratoplasty combined with conjunctival flap covering to treat corneal perforation in severe SJS [19]. However, only anatomic retention was achieved with this procedure as the patients were still blind. SJS usually impairs vision binocularly which reduces a patient’s quality of life and imposes heavy burdens to their families and society. Restoration of sight is vital to such patients.

Based on the severe signs of the ocular complications of SJS such as ocular surface structure abnormality, severe dry eye, corneal opacity, and LSCD, in this study, we concluded the outcomes of corneal sight rehabilitating surgery for the management of ocular complications of SJS.

## Materials and methods

The study was approved by the Institutional Review Board of Peking University Third Hospital (LM2022458) and was conducted in accordance with the tenets of the Declaration of Helsinki. Informed consent was obtained from all participants included in the study.

### Patients

From March 2013 to July 2023, 24 eyes of 18 SJS patients received 29 surgeries, which includes 9 keratoplasties, 8 Keratolimbal allograft (KLAL) and 12 combined surgeries (keratoplasty and KLAL simultaneously) in Peking University Third Hospital. The patients were comprised of four males and 14 females (mean age, 36.7 ± 17.1 [range, 6–72] years). Their medical records were retrospectively reviewed.

### Surgical techniques

Seventeen surgeries were performed with general anesthesia and twelve were performed with retrobulbar anesthesia. KLAL was firstly performed on the patient with LSCD (Video 1). The superficial fibrovascular membrane over the cornea was removed up to a position approximately 3 to 4 mm posterior to the limbus and the bare sclera was exposed. The corneoscleral rims (12.5/13 mm, external diameter; 8/8.5 mm, internal diameter) were harvested from deceased donors.

If the cornea was opaque or perforated, PKP or ALKP was performed depending on the depth of the opacity and the size of the perforation. The lamellar corneal button, with a diameter 0.25 mm larger than the recipient bed. If the recipient’s bed was perforated, an air bubble was then injected into the anterior chamber following ALKP finished. The full-thickness corneal button was 0.5 mm larger than the bed.

Human amniotic membrane, with the basement membrane side up, was used to cover the cornea and the bared sclera in all eyes except two that had fungal keratitis and bacterial keratitis preoperatively.

Extracapsular cataract extraction (ECCE) and intraocular lens insertion (IOL) were performed simultaneously in two eyes. The resected tissues were subjected to real-time polymerase chain reaction (PCR) examination to test for viruses.

### Regular postoperative therapy

**Fig. 1:**
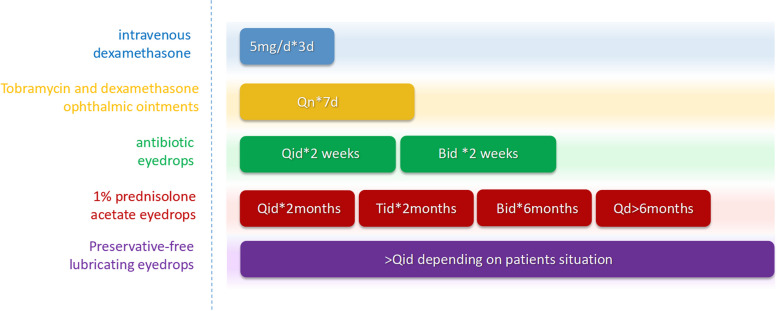
Postoperative routine medication

Postoperative routine medication is shown in Fig. 1. Intravenous glucocorticoid 5 ~ 10mg/d, antibiotic eye drops, glucocorticoid eye drops (except fungal infection), and tacrolimus eye drops were routinely administered for 3 days after surgery. The dosage was gradually reduced according to the patient's condition. The surgeon closely observed the patient's intraocular pressure and corneal repair. The patients were observed daily while they were in the hospital, weekly after leaving the hospital but before the amniotic membrane melted and corneal epithelialization occurred, monthly for the next three months, and trimonthly thereafter. The conjunctival sutures were removed after the amniotic membrane melted. The corneal sutures were removed after they loosened or after more than one year.

### Main outcome measures

VA, optical clarity of the cornea, corneal epithelialization, corneal vascularization, and IOP were reviewed. The lid, conjunctival, and corneal changes at presentation were graded according to the classification of Sotozono et al. [20]. Successful keratoplasty was defined on the basis of corneal epithelialization, decrease in corneal neovascularization, and improvement in VA [21].

### Additional treatment

For patients with persistent epithelial defect or delayed epithelialization, contact lenses were first used to promote epithelialization. If the condition of the cornea did not improve, amniotic membrane was transplanted and 40% diluted (with artificial tears eyedrop) autologous serum eyedrops (four times daily) was administered. If the condition of the cornea still did not improve, permanent tarsorrhaphy was performed. If the cornea had superficial punctate keratopathy, deproteinized calf blood extract eye gel was administered four times per day.

IOP was monitored because of the long-term administration of glucocorticoid eyedrops. If the IOP was above the normal level, IOP-lowering medications were administered at first with or without tapering the dose or concentration of the glucocorticoid eyedrops. If the IOP could not be controlled, external drainage surgery was performed.

If the result of real time PCR of the resected tissues was positive, a topical antiviral drug was administered four times per day over six months. Systemic antiviral medicine was administered for three months in addition to monitoring the results of blood laboratory tests.

If a patient had fungal or bacterial keratitis, anti-fungal or anti-bacterial drugs were administered.

### Statistical analysis

SPSS v25.0 (SPSS Inc. Chicago, IL, USA) was used for statistical analysis. The change in VA was analyzed using the paired t test. The main outcomes of the different surgical procedures were assessed using analysis of variance; Tukey’s test was used for multiple comparisons. The Spearman correlation coefficient (two-tailed) was used to evaluate the correlation between the scores of the 13 components and the total score according to the classification by Sotozono et al., logarithm of the minimum angle of resolution (logMAR) VA, corneal epithelization, corneal neovascularization, and corneal epithelial stability. *P* values less than 0.05 were considered statistically significant.

## Results

Twenty-four eyes were included in this study. The average time from the onset of SJS and to the surgical intervention was 11.3 ± 9.4 (range, 0.25–40) years. An overview of the clinical data of the eyes is shown in Table 1.

**Table 1:** Characteristics, preoperative, and postoperative data of the eighteen patients (24 eyes) with Stevens-Johnson syndrome

No	Sex	Age (y)	Eye	SJS (y)	Surgical procedure	Special condition	FU (m)	Visual acuity (logMAR)				Epi (w)	Additional treatments					
								**Pre.SE**	**CE**	**Optimal**	**endpoint**		**SED**	**CL**	**AMT**	**Tarso**	**Anti-virus**	**AG**
1	F	37	L	2	PKP + ECCE + IOL + AMT	PF	124	2.30	0.50	0.40	0.70	8					20m (PO2m, ED2m)	ED
2	F	24	L	6	PKP + KLAL + AMT		55	1.70	1.70	0.20	2.60	6					53m (PO2m, ED2m)	ED
			L	10	PKP + KLAL + ECCE + IOL + AMT	PF, VK	17	2.30	1.70	0.50	0.50	4		☑			1d (PO3m, ED2y)	ED + V
			L	12	ALKP + AMT	PF, VK, melting	13	2.30	0.80	2.00	2.60	9					1d (ED12m)	
3	F	6	R	1	ALKP + AMT	PF	31	2.30	1.10	0.80	2.00	3	☑	☑		11-17m	11m (ED12m)	
			R	4	ALKP + KLAL + AMT		49	2.00	1.10	0.60	0.60	4	☑	☑			3d (ED12m)	
			L	5	ALKP + KLAL + AMT	VK	60	1.40	0.90	0.50	0.50	4	☑	☑			3d (ED12m)	
			R	8	ALKP + AMT		13	0.90	0.60	0.40	0.40	4						
			R	9	PKP + AMT	PF, melting	12	1.20	0.30	1.40	0.80	2	☑			1-4m		
4	F	54	L	5	ALKP + KLAL + AMT	melting	99	2.30	OP	0.70	2.60	12				2-5m		
5	F	39	R	0.25	ALKP	PF, FK	99	2.30	0.20	0.20	0.30	8	☑	☑	☑	4d-13m		
			L	6	PKP + AMT		35	2.30	0.50	2.00	2.00	2			☑			
6	F	11	R	7	ALKP + KLAL + AMT		90	2.30	2.00	0.30	0.30	4	☑	☑				ED
			L	8	ALKP + KLAL + AMT		59	2.00	0.30	2.00	2.00	4	☑	☑			3d (PO3m, ED2y)	
7	F	38	R	30	KLAL + AMT		88	1.70	2.00	0.00	0.30	1					1d (PO3m, ED2y)	ED
			L	31	KLAL + AMT	OA	67	2.00	0.30	0.70	2.00	1						
8	F	32	L	3	KALT + AMT		87	2.00	2.00	0.20	0.20	1						ED
			R	4	KLAL + AMT		70	2.00	0.50	0.20	0.20	1						ED
9	F	49	R	20	ALKP + KLAL + AMT		81	2.30	0.40	1.00	1.30	28	☑	☑	2m	3-6m		
10	M	30	L	15	KLAL + AMT		75	1.40	0.70	0.20	0.20	6		☑				
11	M	51	L	40	KLAL + AMT		73	1.00	1.00	0.50	1.00	8	☑	☑	2.5m			ED
12	F	31	L	20	KLAL + AMT		69	2.00	2.00	0.50	0.50	1		☑	7d		7m (ED1m)	
13	M	18	R	11	ALKP + KLAL + AMT		65	2.30	0.70	1.00	1.00	20	☑	☑		1-15m	7d (PO2m, ED6m)	ED
14	F	23	R	14	ALKP + KLAL + AMT		65	2.30	2.00	0.60	0.60	24	☑	☑		1-12m	1d (ED8m)	
			L	17	ALKP + KLAL + AMT		27	1.70	1.40	1.20	1.20	^a^						
15	F	72	R	8	PKP	BK, FK	63	2.00	0.40	0.50	1.50	24	☑	☑	7d	20d-12m	20d (PO3m, ED2y)	ED
16	F	51	R	14	ALKP + KLAL + AMT		63	2.00	2.00	0.60	0.60	4		☑				ED
17	M	64	R	10	PKP + KLAL + ECCE + IOL + AMT	PF	58	2.60	1.40	0.20	0.20	4						ED
18	F	31	R	7	KLAL + AMT		50	2.00	0.80	2.00	2.00	3		☑			1d (PO3m, ED6m)	

^a^No record; No. = patient number, *S* Sex, *y* Year, *SJS* Time from onset of Stevens-Johnson syndrome, *pre-VA* Preoperative visual acuity, *FU* Follow-up, *m* Months, *logMAR* Logarithm of the minimum angle of resolution, *pre.SE* Preoperative surgical eye, *CE* Contralateral eye, *Epi (w)* Epithelialization time (weeks), *SED* Serum eye drop, *CL* Contact lens, *AMT* Amniotic membrane transplantation, *Tarso* Tarsorrhaphy, *AG* Anti-glaucoma, *F* Female, *M* Male, *L* Left, *R* Right, *PKP* Penetrating keratoplasty, *ALKP* Anterior lamellar keratoplasty, *KLAL* Kerato-limbal allograft, *ECCE* Extracapsular cataract extraction, *IOL* Intraocular lens insertion, *PF* Perforation, *VK* Virus keratitis, *FK* Fungal keratitis, *OA* Optic atrophy, *BK* Bacterial keratitis, *d* Day, *OP* Ocular prosthesis, *PO* Per os (oral administration), *ED* Eye drop, *V* Valve

The mean preoperative VA was 1.96 ± 0.43 logMAR. The mean fellow VA was 1.05 ± 0.65 logMAR; one patient had an ocular prosthesis in one eye. Fourteen patients had low vision (VA > 0.5 logMAR for both eyes). Nine patients were blind (VA > 1.3 logMAR for both eyes).

Preoperative medications included topical 1% prednisolone acetate (*n* = 7), 1% loteprednol (*n* = 4), 0.1% fluorometholone (*n* = 12), oral prednisone tablets (*n* = 2), topical tacrolimus (*n* = 18), topical amphotericin (*n* = 2), anti-glaucoma eyedrops (*n* = 4), and anti-viral drugs (*n* = 3).

### Surgical procedure

**Fig. 2:**
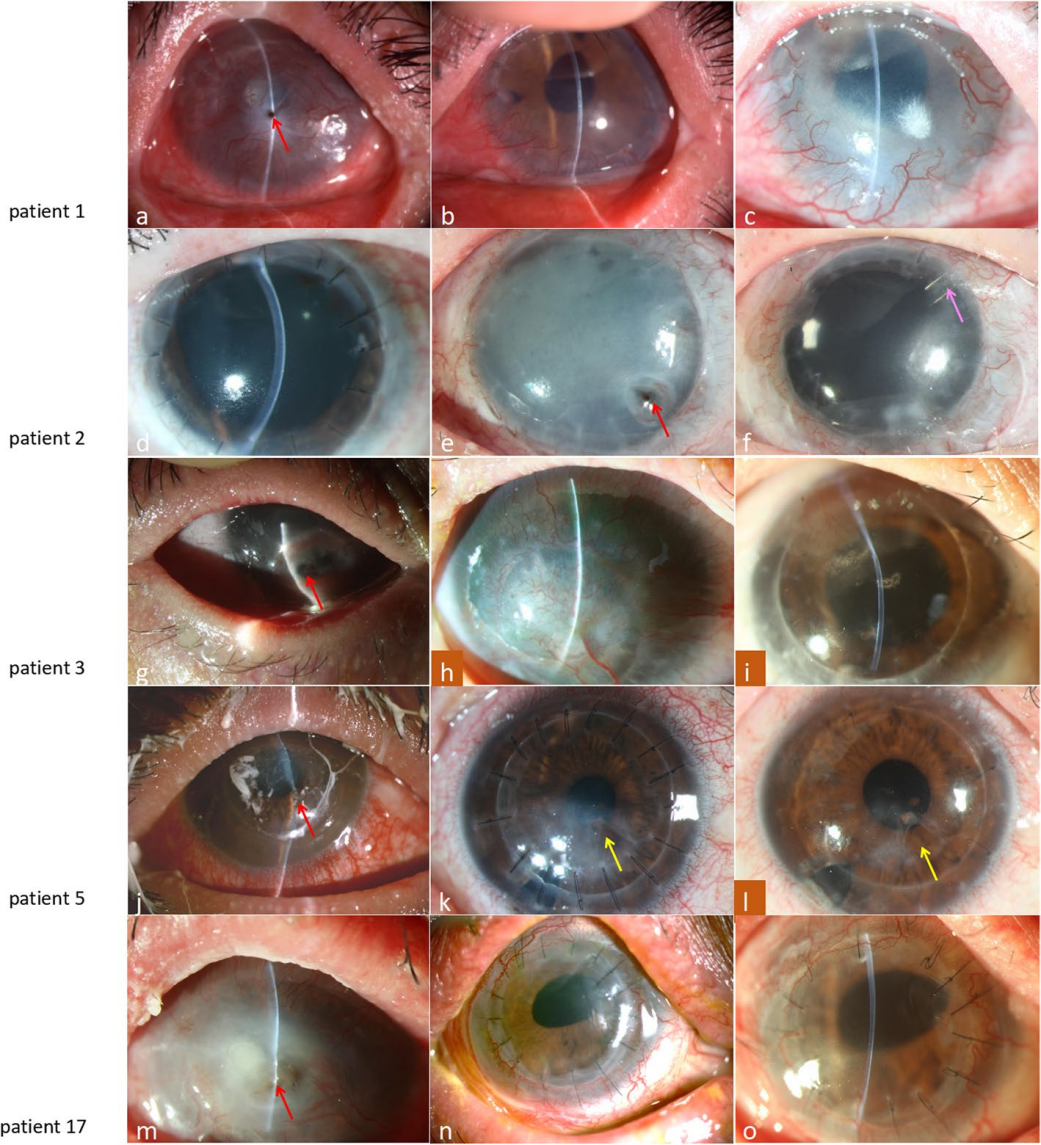
The anterior segment photographs of five patients with corneal perforation. **a**–**c** Patient 1 had corneal perforation on her left eye (**a**. red arrow). The center of the cornea was transparent initially (**b**. two years after surgery), but the transparency gradually decreased (**c**. 6.5 years after surgery). The neovascularization of the cornea tended to be stable; (**d**–**f**) Patient 2 underwent PKP + KLAL + AMT on her left eye with pretty good results for four years (**d**. six months after surgery). The cornea turned opaque and started melting and eventually perforated (**e**. red arrow) 4.5 years after the first keratoplasty because of CMV infection. The second keratoplasty was performed on a hitherto stable ocular surface (**f**). The intraocular pressure could not be controlled with eyedrops; therefore, the Ahmed glaucoma valve (**f**. pink arrow) was inserted 2.5 months after surgery; (**g**–**i**) Patient 3 (a 6-year-old girl) had corneal perforation on her right eye (**g**. red arrow) one year after the onset of SJS. The graft survived but the patient’s visual acuity decreased gradually because of corneal opacity and neovascularization (**h**. 2.5 years after surgery). The second keratoplasty was performed on a stable ocular surface (**i**. 2.5 years after surgery); (**j**–**l**) Patient 5 had fungal keratitis (right eye) in the acute stage of SJS. The cornea was perforated within two days (**j**. red arrow). ALKP was performed to retain her own corneal endothelium. At the early postoperative stage, the graft rapidly became transparent except for the area around the perforation (**k**. two months after surgery). The graft gradually continued to turn transparent and remained stable until the time of this study (**l**. 4 years after surgery). The hole in the Descemet’s membrane can be seen clearly (yellow arrows). **m**–**o** Patient 17 had corneal perforation in his right eye (**m**. red arrow) with negative pathogenic detection. PKP + KLAL + AMT + ECCE + IOL were performed on a hitherto stable ocular surface (**n**. three months after surgery; o. one year after surgery)

**Fig. 3:**
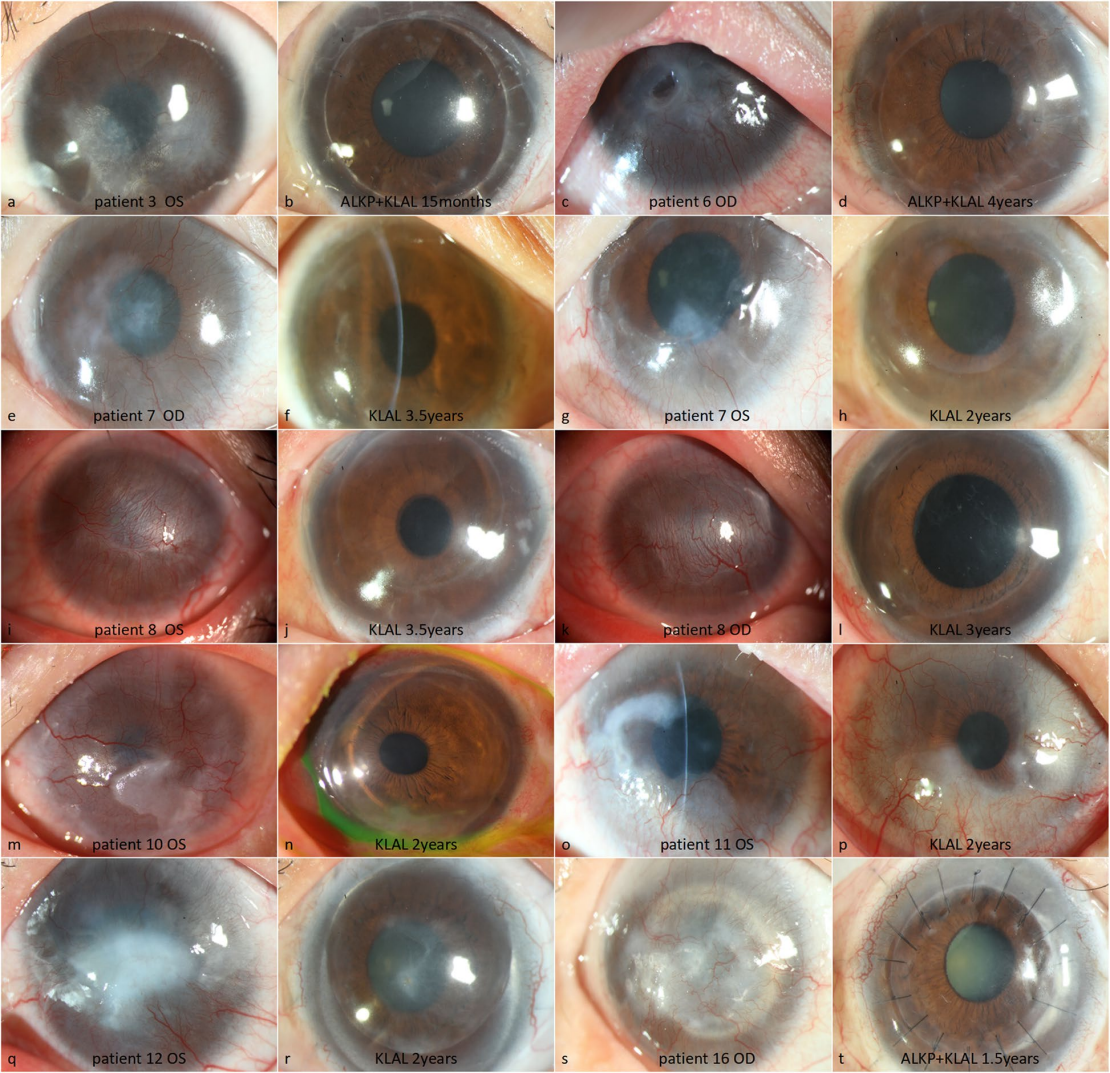
The preoperative and postoperative anterior segment photographs of eyes that had a smooth recovery, stable ocular surface, and relatively transparent central cornea

**Fig. 4:**
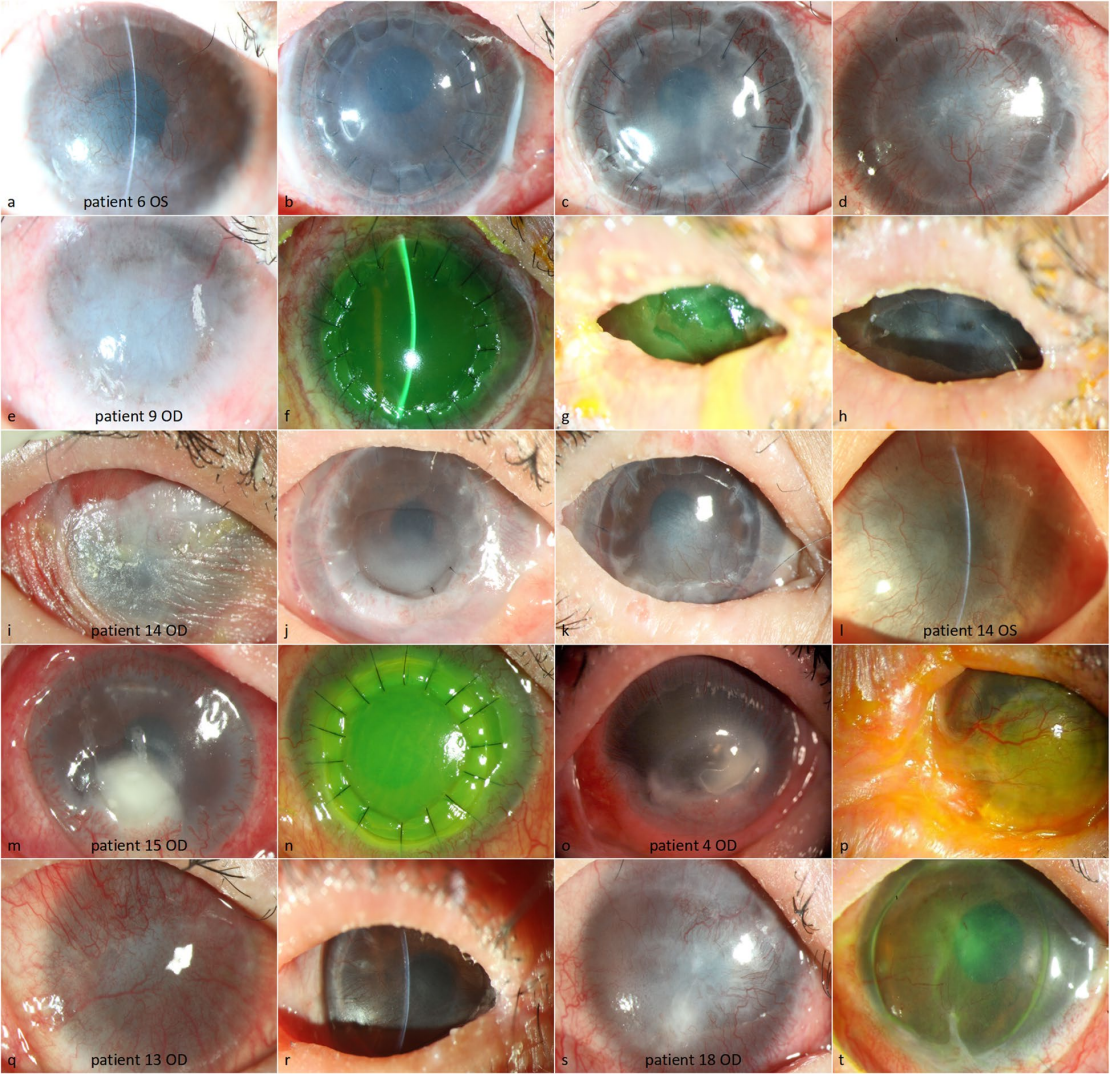
The preoperative and postoperative anterior segment photographs of eyes that had a tortuous recovery process. **a**–**d** Patient 6 underwent anterior lamellar keratoplasty (ALKP), kerato-limbal allograft (KLAL), and amniotic membrane transplantation (AMT) on her left eye because the dissected tissue tested positive for cytomegalovirus (CMV). **a** The graft was transparent and still covered with amniotic membrane 7 days after surgery (**b**). The lamellar graft became progressively opaque and neovascularization occurred (**c**, **d**); (**e**–**h**) ALKP + KLAL + AMT were performed on the right eye of patient 9. The epithelialization did not complete until three months after the surgery (**f**). Tarsorrhaphy was performed (**g**) with a successful epithelialization outcome but a certain degree of corneal opacity remained (**h**); (**i**–**l**) ALKP + KLAL + AMT were performed on the right eye of patient 14, which tested positive for CMV. The ocular surface was keratinized before surgery (**i**). The epithelialization was difficult (**j**) but occurred after tarsorrhaphy from one month to 12 months after surgery. The epithelialization was achieved but a partial corneal opacity remained (**k**). The transparent cornea improved vision and eliminated the patient’s blindness but the contralateral eye had total limbal stem cell defection (**l**); (**m**) Patient 15 had bacterial keratitis on her right eye prior to the surgery. (n) Penetrating keratoplasty was performed for patient 15; (**o**) The right cornea of patient 4 was melting prior to the surgery. **p** The graft was opaque and neovascularization occurred most likely due to poor postoperative management in a local hospital; (**q**, **r**) Patient 13 underwent ALKP + KLAL + AMT on his right eye, which had a stable ocular surface and a relatively transparent cornea that was treated with tarsorrhaphy from one to 15 months after surgery; (**s**, **t**) Patient 13 underwent KLAL + AMT on his right eye, which had a stable ocular surface and a relatively transparent cornea

Twenty-nine surgeries are shown in Table S1. Five surgeries were therapeutic keratoplasty for the treatment of corneal perforation (Fig. 2). The remaining 24 surgeries were optical for visual rehabilitation (Figs. 3 and 4).

### Postoperative treatments

All patients received regular postoperative treatments. Two patients continued oral prednisone tablets for systemic conditions. Two patients used glucocorticoid eyedrops one month later because of prior fungal keratitis. The fungal keratitis patients still received intravenous fluconazole for one week and amphotericin eyedrops for three months. One patient who had bacterial keratitis received intravenous ceftazidime for three days. Six eyes that tested positive for CMV were treated with ganciclovir capsule (three months) and eye gel (more than six months, ranging from six to 18 months).

### Postoperative effects

The mean follow-up period was 50.6 ± 28.1 (range, 12–124) months. The optimal VA (0.74 ± 0.60 logMAR) and endpoint VA (1.06 ± 0.82 logMAR) were both significantly better than the preoperative VA (1.96 ± 0.43 logMAR) (*p* = 0.000). Preoperatively, the visual acuities of fourteen patients were in the low vision spectrum, whereas 9 patients were blind. After surgeries, 57.1% patients (8/14) were no longer in the low vision spectrum, and 88.9% patients (8/9) were no longer blind.

The mean epithelialization time was 7.1 ± 7.6 (range, 1–28) weeks, over 4 weeks in 11 cases, and over 12 weeks in four cases. The success rate was 86.7%. After epithelialization was completed, 13 eyes had a stable corneal epithelium whereas superficial punctate keratopathy was recorded in nine eyes.

Corneal neovascularization developed after 13 surgeries but did not affect central corneal transparency in five eyes. Compared with the preoperative condition of the cornea, the corneal neovascularization of these eyes was alleviated.

The VA and epithelialization times, recorded for the different surgical procedures, were not significantly different (Table S2).

The results of the correlation analyses are shown in Table S3. Preoperative VA was correlated with epithelialization, the loss of palisades of Vogt and conjunctival congestion (*R* = 0.326, *p* = 0.048; *R* = -0.341, *p* = 0.035; *R* = 0.470, *p* = 0.005). Postoperative optimal VA was significantly correlated with postoperative opacification, progressive vascularization, epithelial stability and preoperative neovascularization, opacification and total score (*R* = 0.482, *p* = 0.004; *R* = 0.585, *p* = 0.000; *R* = -0.498, *p* = 0.003; *R* = 0.389, *p* = 0.019; *R* = 0.486, *p* = 0.004; *R* = 0.378, *p* = 0.022). Endpoint VA was significantly correlated with postoperative corneal opacification (*R* = 0.579, *p* = 0.000), progressive vascularization (*R* = 0.575, *p* = 0.000), epithelial stability (*R* = -0.471, *p* = 0.005), and preoperative opacification (*R* = 0.350, *p* = 0.032). Postoperative epithelialization was significantly correlated with postoperative corneal opacification, progressive vascularization, epithelial stability, preoperative corneal opacification, keratinization, conjunctival congestion, trichiasis, meibomian gland in-volvement and total score (*R* = 0. 434, *p* = 0.012; *R* = 0. 601, *p* = 0.000; *R* = -0. 376, *p* = 0.027; *R* = 0. 019, *p* = 0.402; *R* = 0. 437, *p* = 0.011; *R* = 0.703, *p* = 0.000; *R* = 0. 343, *p* = 0.040; *R* = 0.412, *p* = 0.016; *R* = 0.469, *p* = 0.007). Postoperative opacification was significantly correlated with progressive vascularization, epithelial stability, preoperative corneal neovascularization, opacification, trichiasis and total score (*R* = 0.434, *p* = 0.012; *R* = 0.562, *p* = 0.000; *R* = -0.622, *p* = 0.000; *R* = 0.473, *p* = 0.005; *R* = -0.356, *p* = 0.029; *R* = 0.387, *p* = 0.019; *R* = 0.365, *p* = 0.026). Progressive vascularization was significantly correlated with epithelial stability, conjunctival con-gestion, skin mucous involvement, meibomian gland involvement, punctual damage and total score. (*R* = -0.394, *p* = 0.017; *R* = 0.433, *p* = 0.009; *R* = 0.372, *p* = 0.023; *R* = 0.639, *p* = 0.000; *R* = -0.455, *p* = 0.007; *R* = -0.542, *p* = 0.001). Postoperative epithelial stability was significantly correlated with preoperative corneal opacification, keratinization, symblepharon, trichiasis, and total score (*R* = -0.578, *p* = 0.000; *R* = -0.359, *p* = 0.028; *R* = -0.350, *p* = 0.031; *R* = -0.550, *p* = 0.000; *R* = -0.346, *p* = 0.033).

### Management of complications

Ten eyes were treated with serum eyedrops to improve epithelialization and ocular surface stability. Fifteen eyes were treated with therapeutic contact lens. Six eyes underwent one more AMT. Eight eyes underwent permanent tarsorrhaphy to prevent corneal melting because of persistent epithelial defect. The details are presented in Table 1.

Four eyes had glaucoma prior to the surgery. IOP was controlled in 11 eyes with anti-glaucoma eyedrops and adjustment of glucocorticoid eyedrops. IOP was controlled in one eye by insertion of an Ahmed glaucoma valve combined with administration of anti-glaucoma eyedrops. Three eyes had severe allograft opacity; four eyes had a CMV infection before the surgery (Fig. 4 a–d) whereas two eyes underwent delayed epithelialization (Fig. 2g and h and 4o and p). One graft was dysfunctional and melted because of CMV infection (Fig. 2e). Only one graft rejection was observed.

## Discussion

### Sight rehabilitation

In the present study, we analyzed and summarized the long-term outcomes of treating the ocular complications of SJS after corneal sight rehabilitating surgery. Fourteen patients had low vision and 9 patients were blind. Keratoplasty combined or not with KLAL (according the limbal station) was performed on them to rehabilitate their vision. However, many experts do not recommend keratoplasty for such patients because most studies have reported poor outcomes in such cases; these poor outcomes were attributed to the abnormality of the ocular surface and severe dry eye [21–23]. A summary of the previously reported outcomes of cadaveric keratoplasty is presented in Table S4. The rate of graft success was low especially for PKP and ALKP, both of which always needed to be performed two or more times [12, 19, 24–26]. Therefore, most experts concluded that performing PKP for such eyes was futile and should only be performed if extremely necessary. For such severe cases, keratoprosthesis is considered more suitable [11, 22]. However, keratoprosthesis is a high-risk and complex option. The complications of Boston keratoprosthesis is being development of retroprosthetic membrane, elevated IOP, and infectious endophthalmitis [27–29]. The complications of osteo-odonto-keratoprosthesis (OOKP) are vitreous hemorrhage (0–52%), glaucoma (7–47%), endophthalmitis (2–8%), mucosal overgrowth or ulceration, and extrusion of the OOKP [30]. The anatomic retention surgery could not effectively improve the patient’s quality of life, especially in cases of bilateral involvement, which is common in SJS [19]. In our study, before proceeding with keratoplasty or ocular surface reconstruction, we first stabilized the patient's ocular surface through the use of anti-inflammatory and lubricating agents to maintain a relatively stable condition. Subsequently, the appropriate surgical procedure was selected based on the patient's specific eye condition, with particular emphasis on addressing limbal stem cell dysfunction through corneal limbal stem cell transplantation to ensure complete epithelialization post-surgery. Following the operation, tacrolimus eye drops and prednisone acetate eye drops were initiated from the first day to provide potent anti-inflammatory and anti-rejection treatment. It was also important to maintain eye surface moisturization post-surgery and to closely monitor and promptly address any complications that may arise, such as glaucoma or issues related to corneal epithelial healing. There was bilateral involvement in all cases in our study. Both the optimal VA and endpoint VA of the patients improved. After keratoplasty combined or not with KLAL, 57.1% patients (8/14) were no longer in the low vision spectrum, and 88.9% patients (8/9) were no longer blind. A stable corneal epithelium was achieved in 13 eyes, whereas superficial punctate keratopathy was observed in nine eyes. The success rate of the present study was 86.7%. Although postoperative optimal VA was significantly correlated with postoperative opacification, progressive vascularization, epithelial stability, preoperative neovascularization, opacification and total score, we cannot determine the strength of the correlation between parameters due to almost *R* values are < 0.5. Therefore, keratoplasty combined or not with KLAL for treating ocular complications of SJS is a viable option for visual rehabilitation and anatomic retention. The VA was related with postoperative corneal opacification, progressive vascularization, epithelial stability, and preoperative opacification.

### Choice of the operative methods

The surgical procedure for each patient is shown in Table 1. The surgical procedures included single PKP, ALKP, KLAL, and combined surgery depending on the condition of the ocular surface, not just single limbal stem cell or lamellar keratoplasty as was performed in previous reports [6, 19, 31, 32]. In our study, SJS impaired both eyes of the patients, which resulted in stem cells with diminished viability for autologous corneal limbal stem cell transplantation. All the patients who needed limbal stem cell transplantation (LSCT) had total LSCD. Harvesting enough limbal stem cell tissue from living-related donors is very difficult. Moreover, the outcome of living-related LSCT has been reported to be unsatisfactory in such eyes [33–35]. Therefore, all LSCT performed in our study were cadaveric KLAL. Patients affected by SJS are usually young [1]; therefore, we determined that PKP should be avoided unless absolutely necessary to achieve long-term graft survival. ALKP could be attempted even in cases of corneal perforation such as that of patient 5 (Fig. 2 j–l). ALKP + ECCE + IOL + KLAL + AMT rather than PKP + ECCE + IOL + AMT should be performed on patient 1 because we did not have adequate relevant experience at that time. Due to the presence of persistent inflammation and severe dry eye, AMT was used in combination with keratoplasty to promote epithelialization for all eyes with no bacterial or fungal infections.

### Postoperative care and complications management

The ocular surface of the eyes of SJS patients manifests corneal neovascularization and persistent inflammation [36]. The paramount treatment is anti-rejection therapy. The most common immunosuppressor used in previous reports was oral and topical cyclosporine (CSA) (Table S4) [12, 26]. Some patients received systemic cyclophosphamide and mycophenolate mofetil [12, 37]. Plasma concentration needs to be monitored when systemic immunosuppressors are used [12]. Long-term administration of systemic immunosuppressors can produce adverse effects such as increase in serum cholesterol levels, increased risk of infection, and hypomagnesemia [38]; even so, acute rejection can still occur [36]. Therefore, a high rate of rejection may be related to weak immunosuppression with or without CSA. Tacrolimus eyedrop has been available in recent years which has a stronger immunosuppressive effect than CSA eyedrops [39]. All patients in the present study were treated with tapered doses of tacrolimus eyedrop after surgery, without combining them with long-term systemic immunosuppressors, except two patients who used oral prednisone postoperatively for treatment of systemic conditions. All patients were administered with intravenous dexamethasone 5 mg daily for three days to suppress any inflammatory response. Only one graft rejection was observed in all patients. Therefore, we recommend the use of tacrolimus eyedrop to reduce preoperative and postoperative inflammation to increase the graft survival rate.

For such eyes, the poor condition of the ocular surface and LSCD will lead to difficult epithelialization. In the present study, the mean epithelialization time was 7.1 ± 7.6 weeks, and was correlated with preoperative corneal opacification, keratinization, conjunctival congestion, trichiasis, meibomian gland involvement and total score, which led postoperative corneal opacification, progressive vascularization, epithelial defected. Previous studies reported that the epithelialization duration was three days to five months (Table S4). In our study, almost all the procedures were combined with AMT to improve epithelialization. However, the amniotic membrane did not melt within one week that prevented observation of epithelium. Therefore, the epithelialization time recorded was longer than the real duration of epithelialization. In the present study, if epithelialization was delayed, some additional treatments such as serum eyedrops, repeated AMT, therapeutic contact lens, and permanent tarsorrhaphy, were applied to improve epithelialization; some of these therapeutic options were used in previous studies [18, 40]. Therefore, apart from administration of tacrolimus eyedrops, the next essential point for a favorable outcome is to take actions to improve epithelialization on time to prevent corneal melting and occurrence of infective keratitis, which were main reasons for failure in previous studies (Table S4).

In the present study, 1% prednisolone acetate eyedrop was regularly administered to the patients for at least 16 months. Therefore, the occurrence of glaucoma was related to the application of glucocorticoid eyedrops [18, 40]. The IOP of nine eyes was controlled by the administration of anti-glaucoma eyedrops and reduction in the dose of the glucocorticoid eyedrops.

The graft in one eye was dysfunctional and melted due to CMV infection, which may be related to long-term application of tacrolimus eyedrop; however, there is no definite evidence to support this. Five eyes with a severe opaque allograft had CMV infection before the surgery, which may have led to postoperative infection relapse after the use of tacrolimus and prednisolone acetate eyedrops. Therefore, CMV infection should be considered if the graft becomes opaque. Two other cases of severe opaque allografts may be related to delayed epithelialization since both of the patients underwent tarsorrhaphy. Only ALKP could be performed in one case; PKP + KLALT could not be performed because of the lack of a fresh donor full-thickness and limbal stem cell grafts. The other was because the patient was followed up in the local hospital where the staff lacked enough experience in effective management of complications.

## Conclusions

Keratoplasty and KLAL can remarkably enhance VA and improve low vision or even eliminate blindness for ocular complications of SJS. The outcome of the surgeries was correlated with the preoperative ocular situation and choice of operative methods.

### Supplementary Information

**Supplementary Material 1.**

**Supplementary Material 2.**

**Supplementary Material 3.**

**Supplementary Material 4.**

**Supplementary Material 5.**

## Data Availability

The data underlying this article will be shared on reasonable request to the corresponding author.
