# Supplementary material for: The outcomes of corneal sight rehabilitating surgery in Stevens-Johnson syndrome: case series
Source: BMC Ophthalmol. 2024 May 6;24:205. doi: 10.1186/s12886-024-03461-2 (PMC11071215; doi:10.1186/s12886-024-03461-2)
Supplement: Supplementary file 1 — Supplementary Material 1. [file 12886_2024_3461_MOESM1_ESM.doc]

| **Table S1. Surgical procedure** | |
| --- | --- |
| Surgical procedures | Case number |
| PKP | 1 |
| PKP+AMT | 2 |
| PKP+ECCE+IOL+AMT | 1 |
| PKP+ KLAL+AMT | 1 |
| PKP+ KLAL+ECCE+IOL+AMT | 2 |
| ALKP | 1 |
| ALKP+AMT | 3 |
| ALKP+KLAL+AMT | 10 |
| KLAL+AMT | 8 |
| PKP=penetrating keratoplasty; ECCE= extracapsular cataract extraction; IOL=intraocular lens insertion; AMT=amniotic membrane transplantation; KLAL=keratolimbal allograft; ALKP=anterior lamellar keratoplasty. | |
