# Supplementary material for: The outcomes of corneal sight rehabilitating surgery in Stevens-Johnson syndrome: case series
Source: BMC Ophthalmol. 2024 May 6;24:205. doi: 10.1186/s12886-024-03461-2 (PMC11071215; doi:10.1186/s12886-024-03461-2)
Supplement: Supplementary file 2 — Supplementary Material 2. [file 12886_2024_3461_MOESM2_ESM.doc]

| **Table S2. The main outcomes of the different surgical procedures** | | | | | | |
| --- | --- | --- | --- | --- | --- | --- |
|  | **PKP** | **ALKP** | **KLAL** | **PKP+KLAL** | **ALKP+KLAL** | **P value** |
| Number | 4 | 4 | 8 | 3 | 10 |  |
| Preoperative VA (logMAR) | 1.95±0.52 | 1.95±0.70 | 1.76±0.38 | 2.20±0.46 | 2.06±0.31 | 0.555 |
| Follow-up months | 58.5±48.4 | 39.0±40.9 | 72.4±12.0 | 43.3±22.9 | 65.8±20.5 | 0.266 |
| Optimal VA (logMAR) | 1.08±0.76 | 0.85±0.81 | 0.54±0.63 | 0.30±0.17 | 0.85±0.49 | 0.400 |
| Endpoint VA (logMAR) | 1.25±0.61 | 1.33±1.15 | 0.80±0.79 | 1.10±1.31 | 1.07±0.73 | 0.856 |
| Epithelialization (weeks) | 9.0±10.4 | 6.0±2.9 | 2.8±2.8 | 4.7±1.2 | 11.6±9.9 | 0.168 |
| VA=visual acuity; logMAR=logarithm of the minimum angle of resolution; PKP=penetrating keratoplasty; ALKP=anterior lamellar keratoplasty; KLAL=keratolimbal allograft. | | | | | | |
