# Supplementary material for: The outcomes of corneal sight rehabilitating surgery in Stevens-Johnson syndrome: case series
Source: BMC Ophthalmol. 2024 May 6;24:205. doi: 10.1186/s12886-024-03461-2 (PMC11071215; doi:10.1186/s12886-024-03461-2)
Supplement: Supplementary file 3 — Supplementary Material 3. [file 12886_2024_3461_MOESM3_ESM.doc]

| **Table S3. The correlation analyses of the preoperative and postoperative ocular statuses of the patients** | | | | | | | |
| --- | --- | --- | --- | --- | --- | --- | --- |
|  | **Preoperative VA** | **Optimal VA** | **Endpoint VA** | **Epithelialization** | **Postoperative**  **opacification** | **Progressive**  **vascularization** | **Epithelial stability** |
| Epithelialization | 0.048 (0.326)* | 0.212 | 0.073 |  | 0.012 (0.434)^*^ | 0.000 (0.601)^*^ | 0.027 (-0.376)^*^ |
| Postoperative  Opacification | 0.258 | 0.004 (0.482)* | 0.000 (0.579)^**^ | 0.012 (0.434)^*^ |  | 0.000 (0.562)^**^ | 0.000 (-0.622)^**^ |
| Progressive vascularization | 0.111 | 0.000 (0.585)^*^ | 0.000 (0.575)^**^ | 0.000 (0.601)^**^ | 0.000 (0.562)^**^ |  | 0.017 (-0.394)^*^ |
| Epithelial stability | 0.244 | 0.003 (-0.498)* | 0.005 (-0.471)^**^ | 0.027 (-0.376)^*^ | 0.000 (-0.622)^**^ | 0.017 (-0.394)^*^ |  |
| The loss of palisades of Vogt | 0.035 (-0.341)* | 0.339 | 0.258 | 0.120 | 0.443 | 0.410 | 0.147 |
| Epithelial defect | 0.165 | 0.377 | 0.331 | 0.410 | 0.391 | 0.152 | 0.456 |
| Conjunctivalization | 0.097 | 0.359 | 0.347 | 0.078 | 0.426 | 0.437 | 0.375 |
| Neovascularization | 0.440 | 0.019 (0.389)* | 0.068 | 0.138 | 0.005 (0.473)** | 0.061 | 0.132 |
| Opacification | 0.248 | 0.004 (0.486)* | 0.032(0.350)* | 0.019 (0.402)^*^ | 0.029 (0.356)* | 0.139 | 0.000 (-0.578)** |
| Keratinization | 0.457 | 0.235 | 0.397 | 0.011 (0.437)^*^ | 0.327 | 0.084 | 0.028 (-0.359)* |
| Superficial punctate keratopathy | 0.051 | 0.459 | 0.380 | 0.083 | 0.483 | 0.308 | 0.283 |
| Conjunctival congestion | 0.005 (0.470)^**^ | 0.121 | 0.175 | 0.000 (0.703)^**^ | 0.171 | 0.009 (0.433)** | 0.051 |
| Symblepharon | 0.090 | 0.198 | 0.387 | 0.376 | 0.143 | 0.210 | 0.031(-0.350)^*^ |
| Trichiasis | 0.485 | 0.110 | 0.415 | 0.040 (0.343)* | 0.019 (0.387)* | 0.083 | 0.000 (-0.550)^**^ |
| Skin mucous involvement | 0.335 | 0.374 | 0.220 | 0.113 | 0.329 | 0.023 (0.372)^*^ | 0.230 |
| Meibomian gland involvement | 0.056 | 0.056 | 0.145 | 0.016 (0.412)* | 0.193 | 0.000 (0.639)^**^ | 0.445 |
| Punctal damage | 0.415 | 0.113 | 0.103 | 0.212 | 0.198 | 0.007 (0.455)** | 0.407 |
| Total score | 0.206 | 0.022 (0.378)* | 0.175 | 0.007 (0.469)^**^ | 0.026 (0.365)* | 0.001 (-0.542)^**^ | 0.033 (-0.346)^*^ |
| * p<0.05;  ** p<0.01;  The data is p value with Spearman correlation coefficients in brackets when p<0.05; VA, visual acuity. | | | | | | | |
