# Supplementary material for: The outcomes of corneal sight rehabilitating surgery in Stevens-Johnson syndrome: case series
Source: BMC Ophthalmol. 2024 May 6;24:205. doi: 10.1186/s12886-024-03461-2 (PMC11071215; doi:10.1186/s12886-024-03461-2)
Supplement: Supplementary file 4 — Supplementary Material 4. [file 12886_2024_3461_MOESM4_ESM.doc]

| **Table S4. Summary of the published literature on cadaveric keratoplasty for treatment of the ocular complications of Stevens Johnson syndrome and toxic epidermal necrosis** | | | | | | | | | | | | |
| --- | --- | --- | --- | --- | --- | --- | --- | --- | --- | --- | --- | --- |
| **Study** | **N**  **(SJS)** | **Previous surgeries** | **Surgery** | **Follow-up** | **VA**  **improvement** | **Grafts**  **Success** | **SI** | **TI** | **Graft rejection** | **Other complications** | **Subsequent surgeries** | **Epithelialization (Days)** |
| 1994^32^ | 1 |  | KLAL | 18.5 | 6/16  (total) |  |  |  |  |  |  |  |
| 1996^33^ | 5 |  | KLAL+AMT | 4.8 | 4/5 | 3/5 | CSA | CSA |  |  |  |  |
| 1996^25^ | 4 | PKP(3) | KLAL | 10 | 1/4 | 1/4 | CSA | CSA |  |  | PKP(3) | 9.97 |
| 1998^18^ | 4 | AMT | KLAL(2), KLAL+PKP+tarso.(2) | 10.85 | 4/4 | 1/4 | CSA |  |  | Bacterial ulcer | PKP | 14-28 |
| 2001^17^ | 1 |  | KLAL+PKP+ECCE+IOL |  | 1/1 | 0/1 | CSA | CSA | 1/1 | BK | AMT |  |
| 2002^26^ | 7 | 3*PKP(1), 2*PKP(2), PKP(1), ALKP(1) | KLAL(7)+PKP(3)+AMT(1) | 60 |  | 2/7 | CSA (10/25) | CSA (6/25) | 13/23 (total) | IK(1), BK(1) | IrCLAL(4), 2*PKP (2) | 19.3 (3-45) |
| 2002^41^ | 9 |  | KLAL(7);2*KLAL(1);3*KLAL(1), PKP(2),2*PKP(1),3*PKP(2) | >12 | 29.6%  (total) | 62.2% | CSA |  |  | Glaucoma(10/39),35.9%PED, CME(1),IK(3/39) | AMT(9), lid surgery (10/39), filter surgery (9/39), |  |
| 2007^38^ | 2 |  | Cul-allo(1); KLAL(1) | 48 | 2/2 | 2/2 | CSA+  CPP |  |  |  |  | 3(Cul-allo),  21(KLAL) |
| 2011^12^ | 6 | 2*PKP+KLAL(1) | KLAL(6)+PKP(2)+AMT(5) | 49.5 | 4/6 | 1/6  (16.7) | CSA  /MMF | CSA | 8 times | 9/24 increased IOP, 2/24 corneal melting, 4/24IK | KLAL+15*AMT (1), AMT(2), 2*KLAL(2), 3*KLAL(1) | 3delayed  (5 months) |
| 2014^4^ | 13 |  | PKP(8),  ALKP(5) |  |  |  |  |  |  |  |  |  |
| 2014^4^ | 10 | AMT(6), CF(7), 3*ALKP(1), 2*ALKP(1), PKP(1) | ALKP+CF(10) | 21.1 | 6/10 |  |  | CSA |  | CF retraction(1) | Tarso.(1) |  |
| 2018^6^ | 9 |  | KLAL | 15 |  | 7/9 |  |  |  | conjuctivalization |  |  |
| 2019^36^ | 1 |  | KLAL | 16.8 |  | 1/1 | PNS | FK506 |  |  |  | 15.4 |
| 2023^24^ | 4 |  | KLAL(2), KLAL+PKP(1), KLAL+2*PKP(1) | 29.3 | 4/4 | 3/4 |  |  |  | IK(3/4) | PKP(1) |  |
| SJS=Steven’s Johnson Syndrome; TEN=toxic epidermal necrosis; VA=visual acuity; SI=Systemic immunosuppressor; TI=Topical immunosuppressor; KLAL=kerato-limbal allograft transplantation; AMT=amniotic membrane transplantation; PKP=penetrating keratoplasty; tarso.=tarsorrhaphy; ECCE=extracapsular cataract extraction; IOL=intraocular lens; ALKP=anterior lamellar keratoplasty; Cul-allo=culture allograft; CF=conjunctival flap; TL= trabeculectomy; CSA=cyclosporine; CPP=cyclophosphamide; MMF=mycophenolate mofetil; PNS=prednisolone; FK506=tacrolimus; BK=Bullous Keratopathy; IK=infected keratitis; PED=persistent epithelial defect; CME=cystoid macular edema; Lr-CLAL=living related conjunctival allograft; | | | | | | | | | | | | |
